# Charred Straw as a Surgical Dressing

**Published:** 1898-07-16

**Authors:** 


### CHARRED STRAW AS A SURGICAL DRESSING.

The war in Cuba has drawn attention to various forms of extemporised surgical dressings. As a rule, and when within reach of their bases of supply, there is every reason to believe that both armies will find themselves well supplied with all modern appliances, but occasions may arise in which aseptic dressings may run short, and even in civil practice it may often occur that some great accident in a country district, far from town supplies, may render it a matter of the greatest importance to be able at once to obtain an almost unlimited quantity of aseptic dressing material. Under such circumstances it is well to remember a form for dressing introduced, we believe, by Dr. Z. Kikuzi, of the Imperial Japanese Army, at the time of the Chinese-Japanese war, namely, charred straw. A translation ot the original paper is given in the *Georgia Journal of Medicine and Surgery*, from which we derive the following particulars. Charred straw, as used by Dr. Kikuzi, was the black residue after burning rice straw, but it can also be obtained by burning hollow straw of various kinds. About two-thirds of it consists of ashes, and the rest of partially-burned particles of straw. It is very absorbent, and retains the moisture which it takes up. It is light and soft, and from the mode of its extemporaneous production it is probably more aseptic than ordinary dressings, except, of course, such as have been recently sterilised. It is very cheap, and it can be prepared at short notice at almost any place. In its preparation the straw should be burned with only a limited access of air, as it is not pure straw ashes but charred straw which is wanted. "The best method is to take a large iron kettle, fill it with straw, ignite it, and after the straw has ceased to blaze cover it over closely for a short while." One must not forget to stir well the remains of the straw after it has ceased to blaze in order that there may be no unburned ends to give trouble subsequently in the dressings. The best way of using the material is to have bags made of various sizes out of sterilised gauze. These are to be filled with the charred straw and sewn up. Then they are ready for use. When applied the pads should be about three centimetres thick. Practically, it is a dry charcoal dressing, the charcoal being rendered porous by the mixture of partially-consumed fragments of straw. Charcoal is never a pleasant dressing, but it may be of use in an emergency.

